# Sympathetic Responses to Antihypertensive Treatment Strategies: Implications for the Residual Cardiovascular Risk

**DOI:** 10.1007/s11906-025-01339-2

**Published:** 2025-07-23

**Authors:** Raffaella Dell’Oro, Fosca Quarti-Trevano, Pasquale Ambrosino, Guido Grassi

**Affiliations:** 1 Clinica Medica, IRCCS Ospedale San Gerardo, Monza, Italy; 2 Department of Medicine and Surgery, University Milano-Bicocca, Piazza Ateneo Nuovo 1, Milan, 20126 Italy; 3 Istituti Clinici Scientifici Maugeri IRCCS, Scientific Directorate of Telese Terme Institute, 82037 Telese Terme, Italy

**Keywords:** Sympathetic activity, Muscle sympathetic nerve traffic, Lifestyle interventions, Antihypertensive drug treatment, Renal denervation, Carotid baroreceptor stimulation, Residual cardiovascular risk

## Abstract

**Purpose of Review:**

To examine whether and to what extent lifestyle, pharmacological and device-based therapeutic blood pressure lowering interventions are capable to restore a normal sympathetic cardiovascular function in hypertensive patients.

**Recent Findings:**

Data collected by examining the results of more than 50 studies published during the past years by directly quantifying, via microneurography, the sympathetic nerve traffic responses to non-pharmacological and pharmacological antihypertensive treatment have shown that no normalization of the sympathetic cardiovascular function is achieved. Recently, a study by our group carried out in 219 hypertensive patients under monotherapy or combination drug treatment confirmed these results, by showing that, despite achieving an optimal blood pressure control, antihypertensive treatment fails to restore the normal sympathetic neural function detected in the normotensive healthy subjects.

**Summary:**

The sympathetic nervous system plays a key role in blood pressure regulation and hypertension pathophysiology. Recent findings document its involvement also in determining the blood pressure lowering effects of antihypertensive agents. However, the available data show the inability to achieve during treatment a full sympathetic normalization, a finding which may represent one of the mechanisms responsible for the residual cardiovascular risk of the treated hypertensive patient.

## Introduction

A marked activation of the sympathetic neural influences to the heart and the peripheral circulation has been regarded as a key pathophysiological hallmark of the essential hypertensive state, being detectable in the different hypertensive clinical phenotypes, as well as in the earlier and in the more advanced clinical stages of the disease [1–3]. Evidence has been also provided that this neuroadrenergic abnormality, along with favoring the development and progression of the hypertensive state, triggers several cardiovascular and metabolic adverse effects, contributing to the progression of the hypertension-mediated organ damage and of the high blood pressure (BP) - related cardiovascular complications as well [1–2]. This pathophysiological background represents the rationale for considering in the essential hypertensive patients the sympathetic activation a primary target of the antihypertensive treatment strategies [3].

The present paper will review old and new data on how different BP lowering therapeutic interventions may impact on the neuroadrenergic overdrive in hypertension, comparing the results with those collected in normotensive healthy controls. The investigation was limited to the analysis of the data collected by microneurographic recording of sympathetic nerve traffic, because it allowed to examine in more than 1000 hypertensive patients and normotensive controls whether different therapeutic BP lowering interventions might allow to achieve a full sympathetic normalization. This was done properly recognizing the strengths and limitations of the microneurographic approach which will be mentioned below.

Three main topics will be addressed. First, the main features of the sympathetic cardiovascular overdrive in hypertension and in hypertension-mediated organ damage will be highlighted. We will then examine the effects of different lifestyle, pharmacotherapy and device-based BP lowering interventions on the hypertension-related sympathetic overdrive, showing that no therapeutic approach is capable to restore a normal sympathetic function. New findings by our group comparing data collected in treated hypertensive patients and in normotensive controls will be also examined, providing additional evidence on the lack of normalization of the sympathetic neural function in the treated hypertensive patients. The possible implications of these data in the context of the so-called “residual cardiovascular risk” of the treated hypertensive patient will be finally emphasized.

### The Sympathetic Overdrive in Hypertension

During the past half of the century different methodological approaches to assess sympathetic neural function in human beings, such as the assay of circulating venous plasma levels of the adrenergic neurotransmitter norepinephrine, the radiolabeled regional norepinephrine spillover technique, the power spectral analysis of the heart rate signal and the microneurographic recording of efferent postganglionic sympathetic nerve traffic to the skeletal muscle district (MSNA), have provided conclusive evidence that the sympathetic influences to the heart and the peripheral circulation are markedly potentiated in essential hypertension [1–2]. They have also shown that the neuroadrenergic activation may participate at the development and progression of the high BP state and of the hypertension-mediated organ damage [1–3]. The data collected during the years can be summarized as follows. First, the sympathetic overactivity has been shown to develop in the very early clinical phases of the hypertensive state, being already detectable in the normotensive subjects with family history of hypertension and in those individuals displaying BP values in the high normal range, but still below the cutoff office values of 140/90 mmHg defining normality [4–5]. Second, the neurogenic alteration appears to closely mirror the severity of the hypertensive condition, resulting progressively greater in magnitude from the mild to the moderate and the more severe hypertensive state [6]. Third, sympathetic cardiovascular influences have been shown to be activated in a variety of different clinical hypertensive phenotypes, from masked and white-coat hypertension to hypertension of the young (frequently detected in the context of a hyperkinetic circulation), hypertension of the elderly, difficult-to-control high BP pressure state, true drug-resistant hypertension and pregnancy-induced hypertension [2]. Fourth, in presence of hypertension-mediated organ damage, such as left ventricular diastolic dysfunction, left ventricular hypertrophy, increased arterial stiffness and mild to severe renal dysfunction, the sympathetic activation undergoes a potentiation [1–2].This finding suggests, although indirectly, the active participation of adrenergic neural factors, together with the pure hemodynamic alterations typical of the disease, at the development of the structural and functional cardiovascular abnormalities which represent the hallmarks of the complicated hypertensive state [1, 4]. Fifth, when the essential hypertensive state is associated with the presence of an overweight or an obese state, a metabolic syndrome or a chronic renal failure, the degree of the sympathetic activation appears to be markedly reinforced, with an unfavorable impact on the clinical outcome [1, 4]. Finally, in different cardiovascular disease characterized by a relevant sympathetic activation, such as chronic heart failure, renal failure, acute myocardial infarction or acute post-stroke phase, the detection of a marked sympathetic overactivity has been shown to represent a sensitive marker of a poor prognosis in the medium-term period independently on other concomitant risk factors and confounders [7–11].The evidence available in hypertension on the prognostic value of the sympathetic overdrive is more scanty, however, and mainly based on an indirect marker of sympathetic cardiovascular drive, such as resting heart rate values. Indeed, data collected in essential hypertensive patients displaying elevated heart rate values have shown that resting tachycardia is independently and directly related to an increased risk of developing fatal and non-fatal cardiovascular events [12]. Whether this relationship implies that the sympathetic activation carries in hypertension an adverse independent prognostic impact remains to be seen, given the evidence that heart rate cannot be invariably regarded in hypertension as a faithful marker of the adrenergic cardiovascular drive [13].

### Sympathomodulatory Effects of the BP Lowering Interventions

As mentioned above, assessment of sympathetic cardiovascular drive in humans can be performed by different methodological approaches, each of them displaying specific advantages and potential limitations [13]. For the purposes of the present paper the evaluation of the impact of different antihypertensive therapeutic interventions on sympathetic cardiovascular drive has been based on MSNA for a number of reasons. These include the evidence that the microneurographic recording of MSNA (1) allows to obtain highly reproducible values, even when the technique is performed in different experimental sessions spaced each other by weeks and months [13], (2) it is devoid of any confounding placebo effect [13], 3) it represents a faithful approach to dynamically and selectively evaluate central sympathetic neural outflow, without any confounding interference of peripheral processes such as the neural reuptake or the tissue clearance of the adrenergic neurotransmitter [13], or, in the case of the power spectral method, without the interference of vagal influences [13], 4) it can allow comparison between subjects or patients [13] and (5) it has been employed in a large number of studies (more than fifty) aimed at evaluating the sympathetic responses to lifestyle, pharmacological and device-based BP pressure lowering interventions and examining more than one thousand treated hypertensive patients. The microneurographic technique also has a number of limitations, the most important one being represented by the fact that it allows to assess the sympathetic drive in only one cardiovascular district, the skeletal muscle, whose behavior does not necessarily reflect those characterizing other regional cardiovascular areas [1, 13].

### Lifestyle Interventions

The neuroadrenergic effects of the therapeutic interventions based on corrective lifestyle habits such as physical exercise training and dietary restriction of caloric intake have been evaluated in nine microneurographic studies enrolling a total of 143 patients, 134 of them showing elevated BP values [14–22]. All the hypertensive patients examined maintained during the temporal duration of the lifestyle interventions the antihypertensive drug regimen followed before their enrollment in the study proper.

**Fig. 1:**
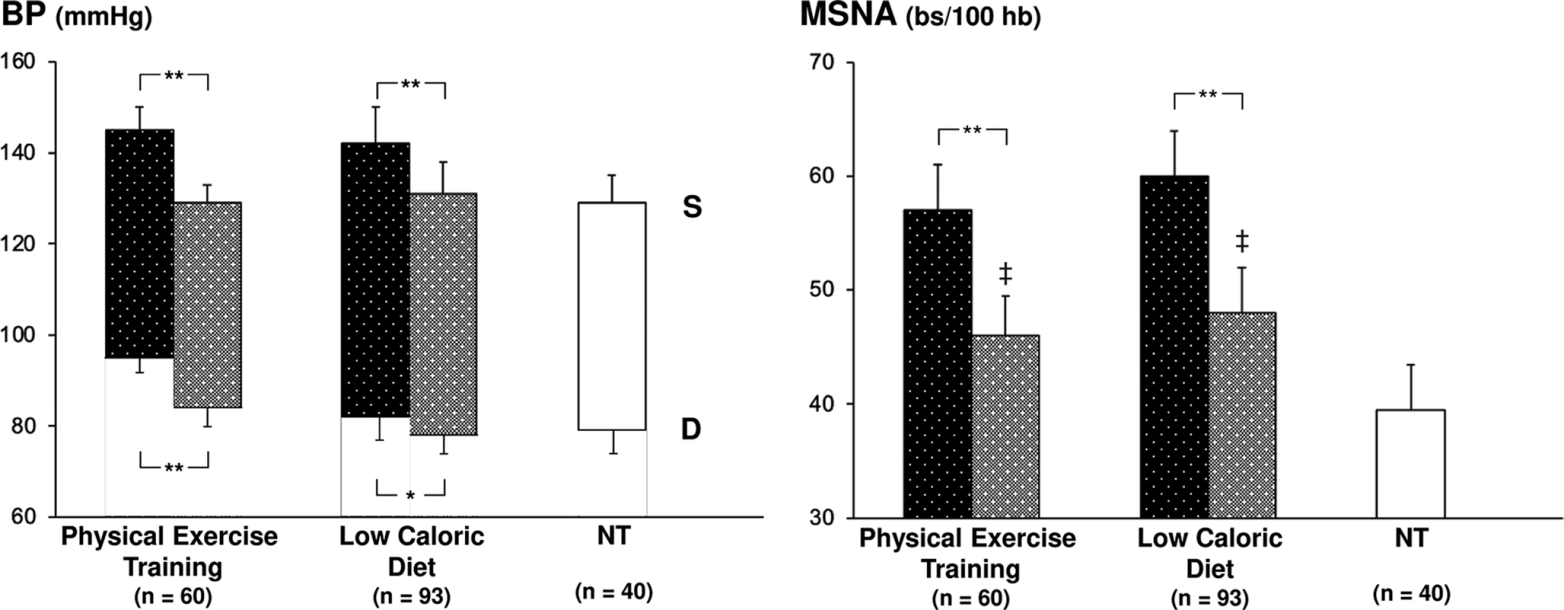
Effects of lifestyle interventions (physical exercise training and low caloric diet program) on systolic (S) and diastolic (D) blood pressure (BP, left panel) and muscle sympathetic nerve traffic (MSNA, right panel) in essential hypertensive patients enrolled in different microneurographic studies [14–22]. Black columns refer to values recorded under baseline condition pre-lifestyle intervention, while grey columns to values recorded at the end of the lifestyle intervention period. A group of age-matched normotensive subjects (NTs) is shown for comparison. Numbers of subjects included in each group are shown in parentheses. Data are shown as means ± standard deviations (SD). Asterisks (*P* < 0.01) refer to the level of statistical significance between values recorded before and at the end of the lifestyle intervention program. The symbol ‡ (*P* < 0.01) refers to the level of statistical significance between values recorded in hypertensive patients at the end of the lifestyle intervention program and values recorded in pure normotensive controls

The effects of physical exercise training programs, lasting between 6 and 16 weeks and consisting in cycling workout weekly sessions, have been assessed in four microneurographic studies involving overall 60 patients, aged between 41 and 71 years (14–17). As shown in Fig. 1, regular physical exercise training triggered, along with a significant office systodiastolic BP reduction, a significant decrease in MSNA. In all the above-mentioned studies the sympathetic responses to physical training were unrelated to (1) the BP lowering effects of the lifestyle intervention and (2) the concomitant changes in resting heart rate values, which not infrequently were negligible in magnitude or even absent [14–17]. Taken together these findings suggest that the BP lowering effects of physical exercise programs are only partially mediated by sympathetic neural mechanisms. They also suggest that the effects of lifestyle interventions on sympathetic function do not appear to be reflected by the heart rate behavior. The latter finding is in line with the observation mentioned above that heart rate does not necessarily reflect, as a marker of adrenergic cardiac drive, the behavior of peripheral sympathetic neural function [13].

The effects of dietary restriction of caloric energy intake on neuroadrenergic function has been evaluated in several studies assessing sympathetic cardiovascular drive with different methodologies [23]. As far as direct recording of sympathetic nerve traffic is concerned, Fig. 1 also shows the results of the 5 microneurographic studies, involving 93 overweight and obese hypertensive patients aged between 52 and 59 years, in which the effects of a hypocaloric diet lasting between 8 to 24 weeks were evaluated on both office BP and MSNA values [18–22]. Systolic BP, diastolic BP and MSNA underwent a significant reduction in the enrolled patients, which showed on average a 10–14% reduction in body weight associated with the hypocaloric dietary intervention. At variance from what it has been already mentioned for the physical training program, the MSNA responses to the diet were significantly and directly related to the BP changes, suggesting a potential ”sympathetic neurogenic nature” of the BP lowering effects of the dietary intervention. Similarly to what already described for physical training, the heart rate reductions associated with the dietary intervention were overall modest in magnitude (on average 3–4 beats/minute) and unrelated to the more pronounced and significant MSNA decreases (on average − 15% to -22% of the baseline pre-intervention values). This latter finding therefore confirms the limitations of heart rate as sensitive marker of the sympathetic modulatory effects of lifestyle interventions mentioned previously.

Finally, Fig. 1 shows the BP (left panel) and MSNA (right panel) values recorded in a group of 40 age-matched pure normotensive healthy individuals. Both the low caloric dietary intervention and the physical training program, although achieving BP values like the ones reported in the normotensive control group, failed to restore a normal sympathetic function. Indeed, the MSNA values detected in the in the group of patients during the lifestyle interventions remained significantly greater than those found in the control group.

### Single Drug Antihypertensive Treatment

**Fig. 2:**
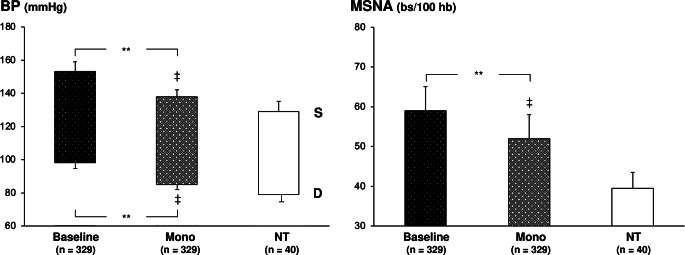
Effects of antihypertensive single drug treatment on systolic (S) and diastolic (D) blood pressure (BP, left panel) and muscle sympathetic nerve traffic (MSNA, right panel) in essential hypertensive patients enrolled in different microneurographic studies [24–43]. Black columns refer to values recorded under baseline no-drug condition (Baseline), while grey columns to values recorded under single drug (Mono) treatment. A group of age-matched normotensive subjects (NTs) is shown for comparison. Asterisks (**P < 0.01) refer to the level of statistical significance between values recorded in the no-drug condition and during treatment. The symbol ‡ (*P* < 0.01) refers to the level of statistical significance between values recorded in treated hypertensive patients and values recorded in pure normotensive controls. For other symbols and abbreviations see preceding figure

During the years 20 have been the published microneurographic studies evaluating the effects of monotherapy with a calcium channel blocker, a central sympatholytic agent, a diuretic, a beta-blocking drug, an ACE-Inhibitor or an angiotensin II receptor blocker on MSNA, for a total of more that 300 patients examined, aged between 43 and 61 years [24–43]. Results, illustrated in Fig. 2, upper panels, show that chronic monotherapy administration, prolonged for a period lasting 1 to 12 weeks accordingly to different studies, induces a significant systodiastolic BP reduction, associated with a significant decrease in resting MSNA values. However, consistent differences were detected between different drugs classes. Indeed while pharmacological compounds acting on beta-adrenergic cardiac receptors or on the renin-angiotensin system (ACE-Inhibitors and Angiotensin II receptor blockers) triggered clearcut and significant sympathoinhibitory effects [24, 26, 28–30, 34, 36–37, 41–42], calcium channels blocking drugs and vasodilating compounds elicited opposite responses, particularly when short-acting dugs were evaluated [25, 33, 35, 43]. Thiazide like diuretics induced almost invariably a potentiation of the sympathetic activation detected in hypertensive patients [36, 38, 40], while anti-aldosterone agents were associated with significant MSNA reductions [38, 40]. These between drug classes differences were also detectable when heart rate values during treatment with different antihypertensive agents were assessed. It should be worthy of mention, however, that (1) the magnitude of the heart rate differences between different drug classes was less pronounced that the one detected via the microneurographic sympathetic nerve traffic recordings and (2) as already reported for lifestyle interventions, no significant relationship was found between the heart rate and the MSNA responses to monotherapy.

Figure 2, upper panels, also reports the BP (left panel) and MSNA (right panel) values recorded in a group of pure normotensive healthy individuals with an age superimposable to the one of the treated hypertensive patients. Antihypertensive treatment based on a single drug administration, despite reducing BP at values similar to the ones reported in the normotensive control group, failed to restore a normal sympathetic function. Indeed, the MSNA values detected in the group of patients under antihypertensive monotherapy remained significantly greater than those found in the control group.

### Combination Drug Therapy

One hundred eighty-nine were the patients, with an age range between 35 and 68 years, evaluated in the 6 published studies examining the effects of combination drug treatment, based on an ACE-Inhibitors plus a calcium channel blocker, an angiotensin II receptor blocker added to a central sympatholytic agent, a thiazide plus an anti-aldosterone drug or an angiotensin II receptor blocker or a calcium channel blocker, on MSNA [37, 44–48]. Results, illustrated in Fig. 2, lower panels, show that chronic combination drug administration prolonged for 4–12 weeks induced a significant systodiastolic BP reduction, amounting on average to 17.0/11.0 mmHg, associated with a significant decrease, on average − 13%, in resting MSNA values. Combination drug treatments based on compounds acting on the renin-angiotensin system and long-lasting calcium channel blockers or central sympatholytic agents are those revealing the most pronounced sympathoinhibitory effects [37, 44, 46]. This finding was confirmed by the evaluation of the heart rate responses to different combination drugs, although, as in the case of monotherapy, no significant relationship was detected between the heart rate and the MSNA responses to different drugs combinations. As previously reported for monotherapy, also in the case of combination drug treatment a comparison was done between the BP and MSNA values recorded in age-matched normotensive controls and those reported in the treated hypertensives (Fig. 2, left and right lower panels). Combination drug treatment, despite reducing BP at values like the ones observed in the normotensive controls, failed to restore a normal sympathetic function. Indeed, the MSNA values detected in the group of patients under pharmacological combination treatment remained significantly greater than those found in the control group.

### Devices-Based Therapeutic Interventions

The recent availability of device-based therapeutic interventions for the treatment of drug-resistant hypertension has allowed to obtain information on the impact of these approaches on BP, hemodynamic variables and sympathetic targets.

The neuroadrenergic effects of bilateral renal nerves ablation have been evaluated in 11 microneurographic studies enrolling a total of 394 drug-resistant and difficult-to-control hypertensive patients [49–59]. Results obtained were also examined in a recent meta-analysis [60]. Age range of the participants was between 53 and 64 years, with an average duration of the follow-up post procedure amounting to 6 months. Results, illustrated in Fig. 4, show that the procedure was accompanied by a significant MSNA reduction, amounting on average to − 8% of the baseline values. This sympathoinhibitory effect was accompanied by a significant systodiastolic BP decrease (-11.4/-5.2 mmHg; *P* = 0.001 for both). No significant quantitative relationship, however, was found between the neuroadrenergic and the BP responses to the procedure, which were not associated with any significant heart rate reduction. More than 10 bilateral renal nerves ablations were needed for obtaining a significant sympathoinhibition.

**Fig. 3:**
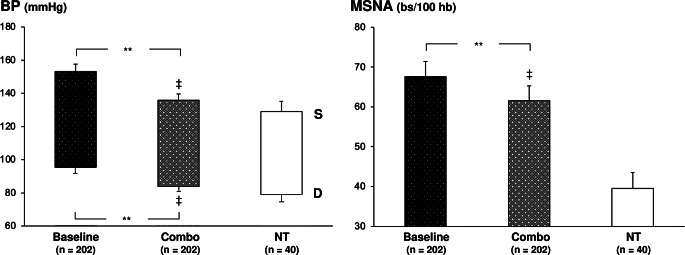
Effects of antihypertensive combination drug treatment on systolic (S) and diastolic (D) blood pressure (BP, left panel) and muscle sympathetic nerve traffic (MSNA, right panel) in essential hypertensive patients enrolled in different microneurographic studies [36, 44–48]. Black columns refer to values recorded under baseline no-drug condition (Baseline), while grey columns to values recorded under combination drug (Combo) treatment. For other explanations and symbols see legend Fig. 2

The other device-based intervention on which information on effects on sympathetic profile have been collected is the acute baroreflex stimulation procedure. Three studies and one recent meta-analysis have provided information on the sympathetic effects of this device-based intervention in 69 resistant hypertensive patients, with an age range between 55 and 67 years, followed during a 1-to-3-month period [61–64]. Results, illustrated in Fig. 3, show that the procedure induced an acute marked and significant reduction in clinic systolic BP (about 20 mmHg, *P* < 0.002), while the diastolic decrease was less marked and non-significant (-5.49 mmHg, *P* = NS]. These BP changes were accompanied by a significant MSNA reduction (-4.28 bursts/min, *P* < 0.05), and by a significant heart rate decrease (-3.65 beats/min, *P* < 0.01). No significant relationship was detected between the MSNA and the systodiastolic BP changes induced by the procedure, this being the case also for the heart rate responses.

### New Evidence and Potential Implications

Studies performed about 30 years ago have shown that the risk of adverse cardiovascular events in hypertensive patients in which treatment is capable to normalize blood pressure values remains more elevated than the one detected in the pure normotensive subjects [65]. This condition, known as “residual cardiovascular risk”, has been suggested to depend on a number of factors, such as (1) the poor control of the risk factors associated with hypertension, (2) the inability of antihypertensive drug treatment to favor a full regression of the hypertension-meditated organ damage, and (3) the failure of effective antihypertensive drug therapy to reduce the elevated BP variability characterizing hypertension [66]. Recently, the hypothesis has been advanced that this condition might depend on the inability of antihypertensive drug treatment to fully restore to normal the heightened sympathetic activation characterizing the hypertensive state [45]. Some indirect supports to this hypothesis have been already mentioned in the previous paragraphs of the present paper, with the evidence that, despite lifestyle, pharmacological or devices-based interventions, sympathetic activity fails fo be normalized by different treatment strategies.

**Fig. 4:**
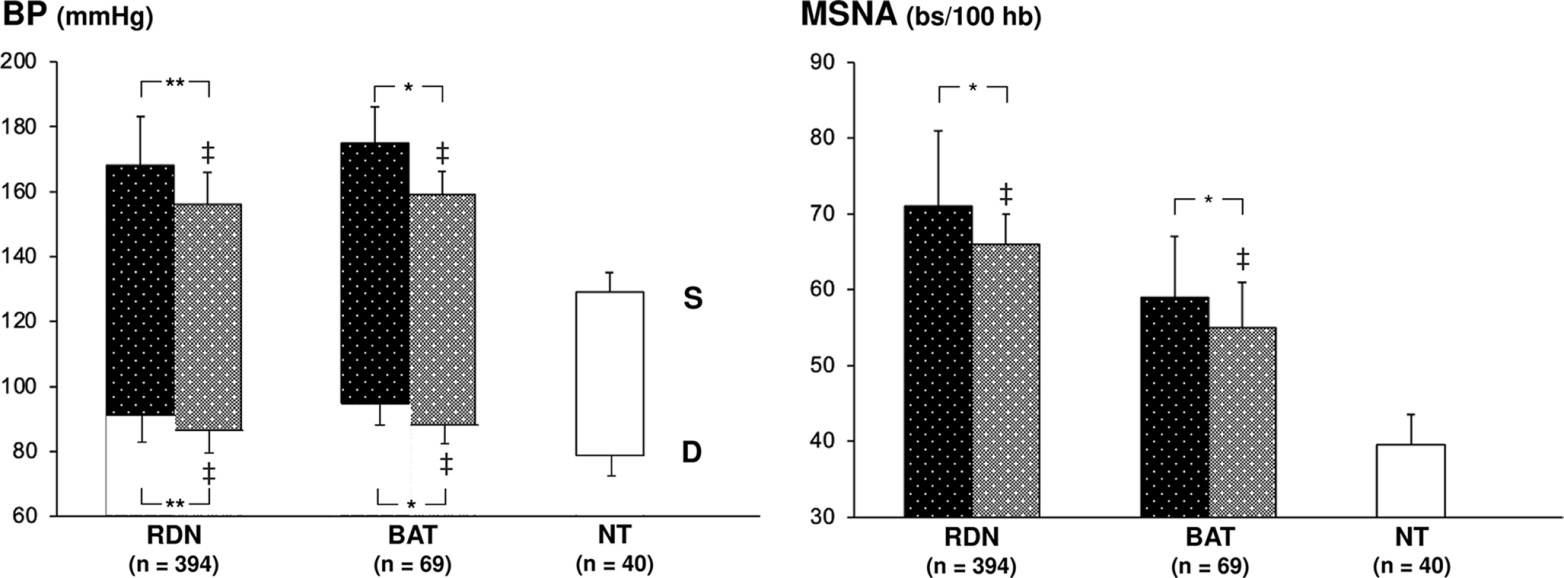
Effects of bilateral renal nerves ablation (RDN) and carotid baroreceptor activation therapy (BAT) on systolic (S) and diastolic (D) blood pressure (BP, left panel) and muscle sympathetic nerve traffic (MSNA, right panel) in resistant hypertensive patients enrolled in different microneurographic studies [49–59]. Black columns refer to values recorded under baseline condition, while grey columns to values recorded under RDN and BAT. Asterisks (*P < 0.05, **P < 0.01) refer to the level of statistical significance between values recorded before and after BAT and RDN. For other symbols and explanations see legends of the preceding figures

**Fig. 5:**
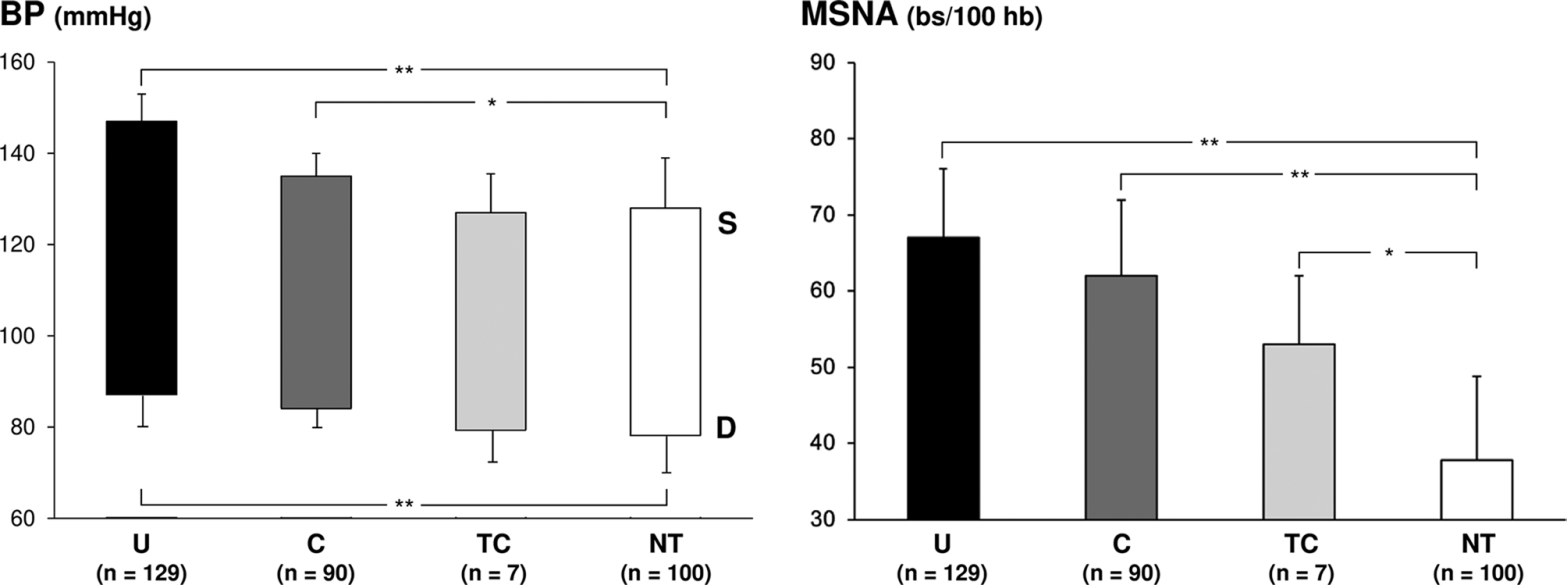
Systolic (S) and diastolic (D) blood pressure (BP, left panel) and muscle sympathetic nerve traffic (MSNA, right panel) in untreated hypertensive patients (U), treated hypertensives with controlled (C) and tightly controlled (TC) BP values. A group of age-matched normotensive subjects (NTs) is shown for comparison. Asterisks (*P < 0.05, **P < 0.01) refer to the level of statistical significance between groups. Data from Ref 67

New evidence supporting the above-mentioned hypothesis come from the results of recent study [67], in which MSNA was assessed in 219 essential hypertensive patients before and after a three months antihypertensive treatment, either as monotherapy or as pharmacological combination of two drugs. To determine the treatment ability to fully restore a normal sympathetic function, results were compared with those obtained in 100 healthy normotensive controls of superimposable age. The results show that despite an effective office blood pressure control induced by treatment, MSNA remained about 65% higher in the treated hypertensive patients as compared to the normotensive controls. This was the case even when treatment was capable to achieve office blood pressure values < 140/90 mmHg or < 130/80 mmHg (Fig. 4). Considering the adverse effects of a persistent sympathetic activation on cardiovascular risk [1, 2], these findings support the hypothesis that one of the mechanisms potentially responsible for determining the residual cardiovascular risk of the treated hypertensive patient is represented by the lack of sympathetic normalization during antihypertensive drug treatment (See Fig. 5).

## Concluding Remarks

The evidence reviewed in the present paper support the concept that the sympathetic overdrive characterizing different hypertensive clinical phenotypes is only in part reversed by non-pharmacological, pharmacological and devices-based interventions, contributing with other factors at determining the residual cardiovascular risk of the treated hypertensive patient. Future studies, based on simplified techniques to assess human sympathetic cardiovascular function, will allow to assess whether and to what extent differences on sympathetic function between different antihypertensive drugs might be responsible for dissimilarities in the clinical outcomes between various BP lowering compounds. They will also allow us to expand the information available so far on the potential role of sympathetic neural factors in modulating patient’s adherence to antihypertensive drug treatment, i.e. a variable of major relevance for determining blood pressure control in treated patients.

## Key References

**2. Grassi G, Dell’Oro R, Quarti-Trevano F, Vanoli J, Oparil S. Sympathetic neural mechanisms in hypertension: recent insights. Curr Hypertens Rep. 2023;28:262 − 70.
  - The paper provides a comprehensive and up-to-date review on the role of the sympathetic nervous system in the development and progression of the essential hypertensive state.
*6. Grassi G, Pisano A, Bolignano D, Seravalle G, D’Arrigo G, Quarti-Trevano F, et al. Sympathetic nerve traffic activation in essential hypertension and its correlates. Systematic reviews and meta-analyses. Hypertension. 2018;72:483 − 91.
  - The meta-analysis examines the data collected in 63 microneurographic studies involving more than 1200 patients showing an almost uniform sympathetic overdrive in the different hypertensive phenotypes.
**45. Fu Q, Zhang R, Witowski S, Arbab-Zadek A, Prasad A, Okazaki K et al. Persistent sympathetic activation during chronic antihypertensive treatment. A potential mechanism for long-term morbidity. Hypertension. 2005;45:513 − 21.
  - This study for the first time advances the hypothesis that the inability of the antihypertensive drug treatment to fully counteract the sympathetic overdrive of the hypertensive state may be involved in the long-term morbidity of the disease.
**65. Andersson OK, Almgren T, Persson B, Samuelsson O, Hedner T, Wilhelmsen L. Survival in treated hypertension: follow-up study after two decades. BMJ. 1998;317:167 − 71.
  - The study represents the first report showing the presence of the “residual cardiovascular risk” in treated hypertensive patients, despite an optimal blood pressure control.
**66. Mancia G, Kreutz R, Brunström M, Burnier M, Grassi G, Januszewicz A, et al. 2023 ESH guidelines for the management of arterial hypertension. J Hypertens. 2023;41:1874–2071.
  - This is the Guidelines document of the European Society of Hypertension issued in 2023, addressing all the clinical and therapeutic aspects of hypertension, including a detailed critical review of the so-called “residual cardiovascular risk”.
*67. Quarti-Trevano F, Seravalle G, Facchetti R, Tsioufis K, Dimitrias K, Manta E, et al. Failure of antihypertensive treatment to restore normal sympathetic activity. Hypertension. 2025;82:1024-34.
  - This recently published study documents for the first time the inability of the antihypertensive drug treatment to provide a full normalization of the sympathetic cardiovascular function in well treated hypertensive patients.

## Data Availability

No datasets were generated or analysed during the current study.
